# Copolymers Based on 1,3-Bis(carbazol-9-yl)benzene and Three 3,4-Ethylenedioxythiophene Derivatives as Potential Anodically Coloring Copolymers in High-Contrast Electrochromic Devices

**DOI:** 10.3390/polym8100368

**Published:** 2016-10-19

**Authors:** Chung-Wen Kuo, Teng-Lu Wu, Yuan-Chung Lin, Jeng-Kuei Chang, Ho-Rei Chen, Tzi-Yi Wu

**Affiliations:** 1 Department of Chemical and Materials Engineering, National Kaohsiung University of Applied Sciences, Kaohsiung 80778, Taiwan; welly@cc.kuas.edu.tw (C.-W.K.); danny116040@yahoo.com.tw (T.-L.W.); chz@cc.kuas.edu.tw (H.-R.C.); 2 Institute of Environmental Engineering, National Sun Yat-Sen University, Kaohsiung 804, Taiwan; yclin@faculty.nsysu.edu.tw; 3 Institute of Materials Science and Engineering, National Central University, Taoyuan 32001, Taiwan; jkchang@ncu.edu.tw; 4 Department of Chemical and Materials Engineering, National Yunlin University of Science and Technology, Yunlin 64002, Taiwan

**Keywords:** electrochromic device, conducting polymer, electrochemical polymerization, optical contrast, coloration efficiency, poly(3,4-ethylenedioxythiophene)-poly(styrene sulfonic acid)

## Abstract

In this study, copolymers based on 1,3-bis(carbazol-9-yl)benzene (BCz) and three 3,4-ethylenedioxythiophene derivatives (3,4-ethylenedioxythiophene (EDOT), 3,4-(2,2-dimethylpropylenedioxy)thiophene (ProDOT-Me_2_), and 3,4-ethylenedithiathiophene (EDTT)) were electrochemically synthesized and their electrochemical and electrochromic properties were characterized. The anodic copolymer P(BCz-*co*-ProDOT) with BCz/ProDOT-Me_2_ = 1/1 feed molar ratio showed high optical contrast (Δ*T*%) and coloring efficiency (η), measured as 52.5% and 153.5 cm^2^∙C^−1^ at 748 nm, respectively. Electrochromic devices (ECDs) based on P(BCz-*co*-EDOT), P(BCz-*co*-ProDOT), and P(BCz-*co*-EDTT) as anodic polymer layers, and poly(3,4-ethylenedioxythiophene)-poly(styrene sulfonic acid) (PEDOT-PSS) as cathodic polymer layer were fabricated. P(BCz-*co*-ProDOT)/triple-layer PEDOT-PSS ECD showed three different colors (light yellow, yellowish-blue, and dark blue) at different applied potentials. In addition, the highest optical contrast (Δ*T*%) of P(BCz-*co*-ProDOT)/triple-layer PEDOT-PSS ECD was found to be 41% at 642 nm and the coloration efficiency was calculated to be 416.5 cm^2^∙C^−1^ at 642 nm. All ECDs showed satisfactory optical memories and electrochemical cyclic stability.

## 1. Introduction

Organic electroactive materials have gained much attention for commercial electronic devices due to their benefits of facile structural modifications, high optical contrast between redox state, and fast photo-switching ability. π-conjugated polymers (CPs) are among the most widely explored organic electroactive materials. CPs have been widely used in advanced technological fields including polymer solar cells [1,2], polymer light-emitting diodes [3,4], catalysts [5,6,7], fluorescent sensors [8], thin film transistors [9], and electrochromic devices (ECDs) [10]. In these fields, researchers have focused enormously on the application of CPs in ECDs due to CPs being able to change their spectroelectrochemical properties after application of an electrical voltage [11].

In the last decade, a class of CPs, known as polypyrroles [12], polythiophenes (PTh) [13], polyanilines [14], polyfurans [15], polycarbazoles (PCz) [16], polyazulenes [17], polyindoles [18], and poly(3,4-ethylenedioxythiophene)-poly(styrene sulfonic acid) [19] have been widely used in electrochromic materials. Among them, PCz is quite bleached in its neutral state and becomes colored in its oxidized state. Carbazole can be substituted or polymerized at the 3,6- or 2,7-positions and a wide variety of alkyl and aryl chains can be incorporated into the nitrogen atom of the carbazole unit, leading it to be a good candidate for a number of opto-electronic applications. PTh and their derivatives have been widely used due to their good redox reversibility, high conductivity value, and the high optical contrast between redox states [20]. PTh can be formed directly on the electrodes using electrochemical polymerization. Poly(3,4-ethylenedioxythiophene)s (PEDOT) and poly(3,4-(2,2-dimethylpropylenedioxy)thiophene) (PProDOT-Me_2_) contain two electron-donating oxygen atoms on the 3,4-positions of the thiophene unit, which decreases the onset potentials of polymer films, and makes the band gaps of PEDOT and PProDOT-Me_2_ films lower than PTh [21]. Moreover, 3,4-ethylenedithiathiophene (EDTT) is an electron-donating heterocyclic unit with two electron-donating sulfur atoms on the 3,4-positions of the thiophene unit, leading to shifts in the onset potential and absorption maximum of the polymer film. Copolymerization of distinct monomers containing several diverse units can give rise to an interesting combination of the electrochromic and electrochemical properties observed in the corresponding homopolymers. For this matter, copolymers based on the carbazole derivative and 3,4-ethylenedioxythiophene derivatives were synthesized electrochemically in this study. Furthermore, 1,3-bis(carbazol-9-yl)benzene contains two carbazole units linked by a phenyl unit, which permits charge carrier transport and eases the formation of stable radical cations (polaron) and dication (bipoloran). Three copolymers (P(BCz-*co*-EDOT), P(BCz-*co*-ProDOT), and P(BCz-*co*-EDTT)) were synthesized using electrochemical copolymerizations, and the spectroelectrochemical and electrochromic properties of copolymer films were systematically and comprehensively studied. It was interesting to find that the slight structural variations of the 3,4-ethylenedioxythiophene derivatives brought about distinct electrochromic properties.

There have been few reports about 1,3-bis(carbazol-9-yl)benzene-based copolymers as anodic layers in ECDs. For this matter, several ECDs were fabricated using PBCz, P(BCz-*co*-EDOT), P(BCz-*co*-ProDOT), and P(BCz-*co*-EDTT) as the anodic layers of the coloring electrode, and poly(3,4-ethylenedioxythiophene)-poly(styrene sulfonic acid) (PEDOT-PSS) as the cathodic layer of the complementary electrode. The spectroelectrochemistry, optical contrast, switching velocity, and cycling stability of the ECDs were also studied.

## 2. Materials and Methods

### 2.1. Materials

2,2-Dimethyl-3,4-propylenedioxythiophene (ProDOT-Me_2_) was prepared based on previously published procedures [22]. 1,3-Bis(carbazol-9-yl)benzene (BCz), 3,4-ethylenedioxythiophene (EDOT), and 3,4-ethylenedithiathiophene (EDTT) were purchased from Sigma-Aldrich (St. Louis, MO, USA). For the materials of polymer composite electrolytes, PMMA (*M*_w_ = 350,000) purchased from Acros organics (Geel, Belgium) was dried at 100 °C under vacuum for 1 day and LiClO_4_ obtained from Aldrich was dried at 120 °C under vacuum for 1 day. Propylene carbonate (often abbreviated PC) was bought from Alfa Aesar (Ward Hill, MA, USA) and was used as received.

### 2.2. Electrochemical Polymerization of PBCz, P(BCz-co-EDOT), P(BCz-co-ProDOT), and P(BCz-co-EDTT) Films

PBCz, P(BCz-*co*-EDOT), P(BCz-*co*-ProDOT), and P(BCz-*co*-EDTT) films were prepared potentiostatically at 1.0 V (vs. Ag/AgNO_3_) on ITO electrodes with a charge density of 60 mC∙cm^−2^, using 0.002 M monomer in a solution containing 0.2 M LiClO_4_ in PC/acetonitrile (ACN) solution. The feed species of these films are summarized in Table 1. An Ag/AgNO_3_ electrode (calibrated using ferrocene) and a platinum wire were used as the reference and counter electrodes, respectively. The double layers cathodic PEDOT-PSS polymer film was prepared using spin coating techniques; the spin condition for film preparation was 2000 rpm. The active area of polymer film on indium tin oxide (ITO) electrode was 1.0 × 1.5 cm^2^.

### 2.3. Construction of Electrochromic Devices

**Figure 1:**
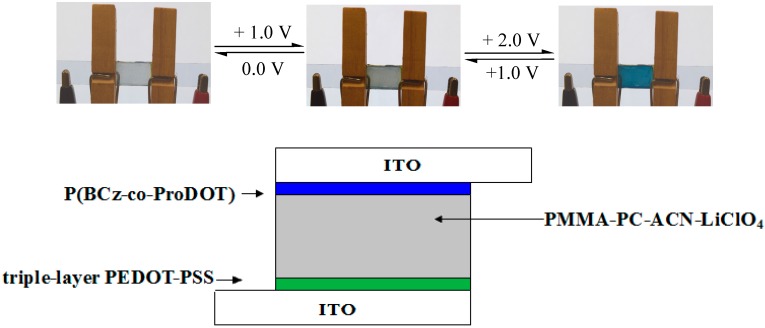
Schematic diagrams of P(BCz-*co*-ProDOT)/triple-layer PEDOT-PSS device.

A gel electrolyte consisting of PMMA, 0.2 M LiClO_4_, PC, and ACN was prepared and the gel polymer electrolyte was coated on anodic PBCz, P(BCz-*co*-EDOT), P(BCz-*co*-ProDOT), and P(BCz-*co*-EDTT) films. A double-layer cathodic PEDOT-PSS film was placed onto the electrolyte membrane to construct an electrochromic device. The edges of electrochromic devices were sealed with epoxy resin to prevent the interface from being attacked by moisture and oxygen. The effective area of the prepared electrochromic devices was about 1.5 cm^2^. For comparison, cathodic films were coated with a triple-layer PEDOT-PSS and a quadruple-layer PEDOT-PSS on ITO electrodes. ECDs were also built by arranging P(BCz-*co*-ProDOT) and a triple-layer PEDOT-PSS (or quadruple-layer PEDOT-PSS) facing each other in order that they could to be separated by a gel electrolyte. The configuration of ECD is shown in Figure 1.

### 2.4. Electrochemical and Spectroelectrochemical Characterization

The electrochemical experiments were executed in a multi-component cell with a CHI627D electrochemical analyzer (CH Instruments, Austin, TX, USA). An ITO coated glass plate (1 × 1.5 cm^2^ area), Ag/AgNO_3_ electrode, and platinum wire were used as working, reference, and counter electrodes, respectively. The spectroelectrochemical experiments were performed with a HITACHI spectrophotometer to record the in situ UV–Visible spectra. The double potential chronoamperometry was carried out with the assembled cell using a CHI627D electrochemical analyzer and a HITACHI spectrophotometer (Hitachi, Tokyo, Japan).

## 3. Results and Discussion

### 3.1. Electrochemical Polymerization

**Figure 2:**
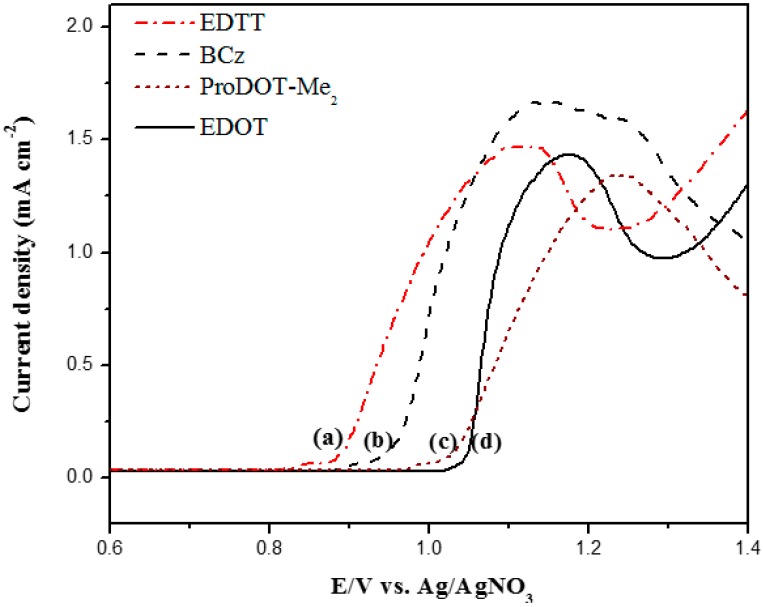
Anodic polarization curves of: (**a**) 2 mM EDTT; (**b**) 2 mM BCz; (**c**) 2 mM ProDOT-Me_2_; and (**d**) 2 mM EDOT in a PC/ACN (1:1, by volume) solution containing 0.2 M LiClO_4_ at a scan rate of 100 mV∙s^−1^.

Figure 2 shows the anodic polarization curves of the neat EDTT, BCz, ProDOT-Me_2_, and EDOT in PC/ACN solution containing 0.2 M LiClO_4_ at a scan rate of 100 mV∙s^−1^. The onset potential of neat EDTT, BCz, ProDOT-Me_2_, and EDOT were 0.86, 0.94, 1.00, and 1.04 V, respectively. The *E*_onset_ of EDTT is smaller than that of EDOT, indicating the incorporation of dithio group on 3,4-positions of thiophene led to lower oxidation potentials of polymer films than the dioxy group. Moreover, EDTT showed lower *E*_onset_ than BCz and ProDOT-Me_2_. The discriminations between onset potential of neat BCz vs. neat EDOT, neat BCz vs. ProDOT-Me_2_, and neat BCz vs. neat EDTT were small (<0.1 V), implying the feasibility of copolymerizations of BCz with EDTT (or ProDOT-Me_2_, EDOT).

**Figure 3:**
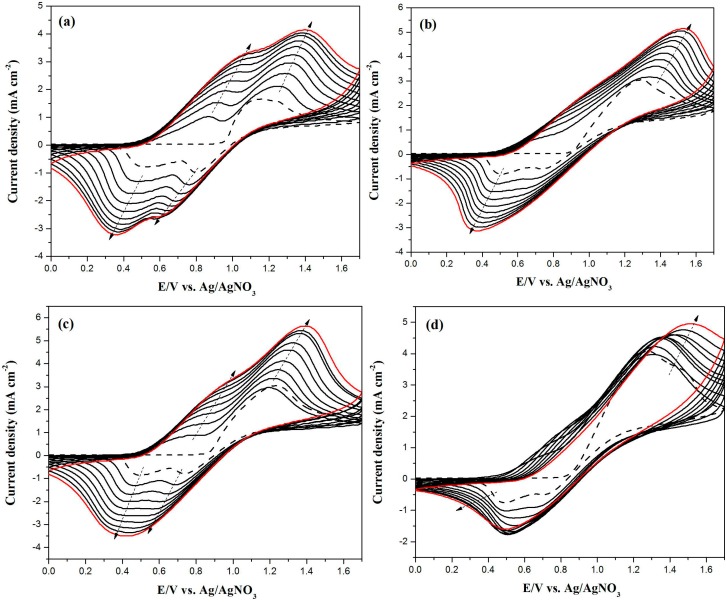
Electrochemical synthesis of: (**a**) PBCz; (**b**) P(BCz-*co*-EDOT); (**c**) P(BCz-*co*-ProDOT); and (**d**) P(BCz-*co*-EDTT) in a PC/ACN (1:1, by volume) solution containing 0.2 M LiClO_4_ at 100 mV∙s^−1^ on ITO working electrode. Arrows, red line

**Figure 4:**
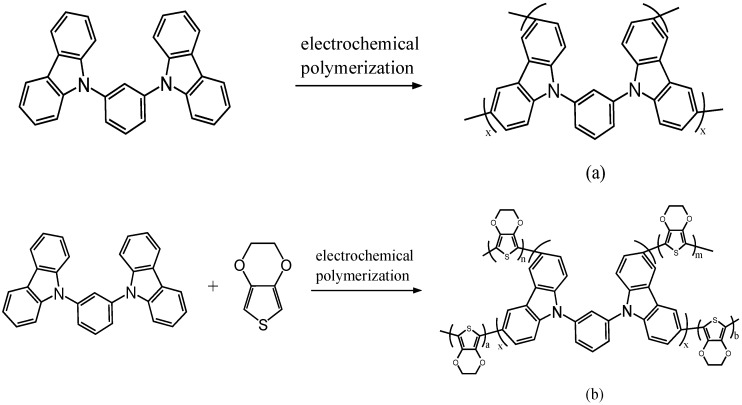

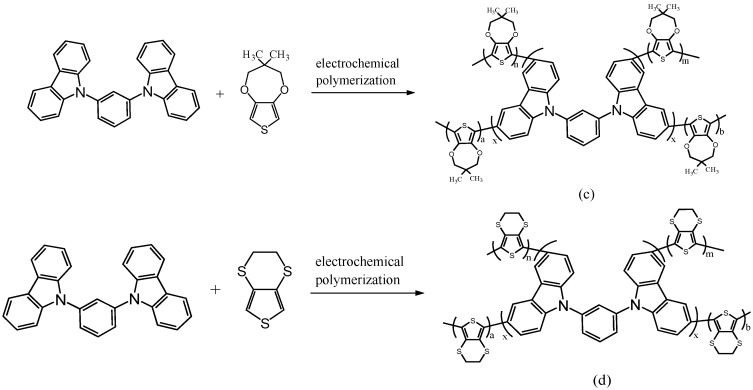
The electrochemical polymerization routes of: (**a**) PBCz; (**b**) P(BCz-*co*-EDOT); (**c**) P(BCz-*co*-ProDOT); and (**d**) P(BCz-*co*-EDTT).

Figure 3a–d shows the cyclic voltammograms of the neat BCz, the mixture of BCz + EDOT, the mixture of BCz + ProDOT-Me_2_, and the mixture of BCz + EDTT in 0.2 M LiClO_4_/(PC + ACN) solution at a scan rate of 100 mV∙s^−1^. As the CV scan continued, the relative intensities of the oxidation and reduction peaks increased with increasing scanning cycles; this can be attributed to the growth of homopolymers and copolymers on the electrode [23]. As shown in Figure 3a, PBCz had two oxidation peaks at 0.87 and 1.25 V and two reduction peaks at 0.46 and 0.75 V. The oxidation and reduction peaks of P(BCz-*co*-EDOT) located at 1.34 and 0.47 V (Figure 3b), which are different to the redox potentials of neat PBCz, demonstrating the formation of P(BCz-*co*-EDOT). In a similar condition, P(BCz-*co*-ProDOT) had two oxidation peaks at 0.79 and 1.19 V and two reduction peaks at 0.45 and 0.69 V (Figure 3c). The oxidation and reduction peaks of the P(BCz-*co*-EDTT) film occurred at 1.32 and 0.50 V (Figure 3d). The redox potentials of P(BCz-*co*-ProDOT) and P(BCz-*co*-EDTT) films were different to those of PBCz, indicating that the copolymer films were deposited on the ITO electrodes. The electrochemical polymerization routes of PBCz, P(BCz-*co*-EDOT), P(BCz-*co*-ProDOT), and P(BCz-*co*-EDTT) are displayed in Figure 4.

**Figure 5:**
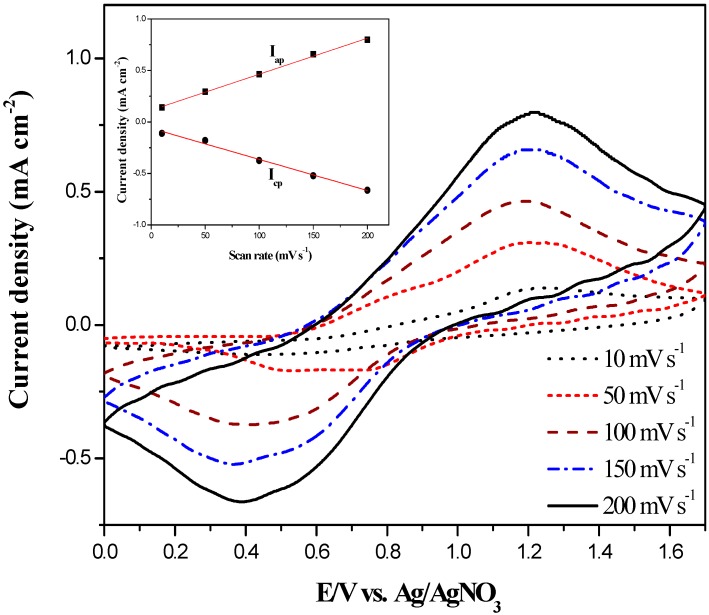
Cyclic voltammetry (CV) curves of the P(BCz-*co*-ProDOT) film at different scan rates between 10 and 200 mV∙s^−1^ in a PC/ACN (1:1, by volume) solution containing 0.2 M LiClO_4_. The inset is scan rate dependence of the P(BCz-*co*-ProDOT) anodic and cathodic peak current densities, respectively.

The P(BCz-*co*-ProDOT) films deposited through the electrocopolymerization of BCz and ProDOT-Me_2_ were studied at various scan rates between 10 and 200 mV∙s^−^^1^ in order to verify the scan rate dependence of the copolymer films. Figure 5 shows the cyclic voltammograms of the P(BCz-*co*-ProDOT) film (prepared by scanning the potentials between 0.0 and 1.7 V) at various scan rates in 0.2 M LiClO_4_/(PC + ACN) solution. The P(BCz-*co*-ProDOT) film shows a couple of oxidation and reduction peaks and the current density of redox peaks is linearly proportional to the scan rate, implying the P(BCz-*co*-ProDOT) film well-adhered onto the ITO electrodes and that the electrochemical processes of P(BCz-*co*-ProDOT) film were characteristic of a nondiffusional redox process [24].

### 3.2. Electrochromic Characterizations of Polymer Films

**Figure 6:**
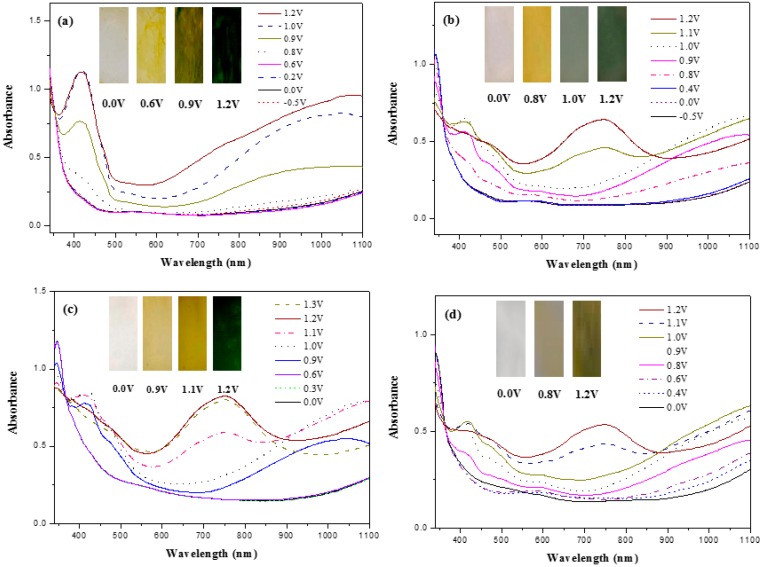
UV–Visible spectra of: (**a**) PBCz; (**b**) P(BCz-*co*-EDOT); (**c**) P(BCz-*co*-ProDOT); and (**d**) P(BCz-*co*-EDTT) on ITO in a PC/ACN (1:1, by volume) solution containing 0.2 M LiClO_4_.

Spectroelectrochemistry of the PBCz, P(BCz-*co*-EDOT), P(BCz-*co*-ProDOT), and P(BCz-*co*-EDTT) films coated on ITO electrode was investigated in 0.2 M LiClO_4_/(PC + ACN) solution. Figure 6a–d shows the spectroelectrochemical spectra of PBCz, P(BCz-*co*-EDOT), P(BCz-*co*-ProDOT), and P(BCz-*co*-EDTT) films, respectively, at various potentials in 0.2 M LiClO_4_/(PC + ACN) solution. There was no conspicuous absorption peaks of PBCz film in the neutral state. Upon applying 0.9 V, new charge carrier bands appeared at 420 and 1050 nm, which can be assigned to the formation of polaron and bipolaron bands [25]. The polaron and bipolaron bands of P(BCz-*co*-EDOT) film in 0.2 M LiClO_4_/(PC + ACN) solution located at 420, 732, and 1050 nm, and at the middle band (732 nm) can be ascribed to the polaron and bipolaron bands of 3,4-ethylenedioxythiophene unit in an oxidation state. Similarly, the polaron and bipolaron bands of P(BCz-*co*-ProDOT) film occurred at 420, 748, and 1050 nm in moderate and high oxidized states, whereas those of P(BCz-*co*-EDTT) film located themselves at 420, 749, and 1050 nm. The absorption peaks of P(BCz-*co*-ProDOT) and P(BCz-*co*-EDTT) at 748 and 749 nm, respectively, can be attributed to the polaron and bipolaron bands of ProDOT-Et_2_ and EDTT units in an oxidation state.

The PBCz film showed multicolor electrochromism, which was transparent in the neutral state (0.0 V), light yellow in the intermediate state (0.6 V), yellowish green in the oxidized state (0.9 V), and blackish green in highly oxidized states (1.2 V). For the copolymer films, the P(BCz-*co*-EDOT) film was transparent in the neutral state (0.0 V), yellow in the intermediate state (0.8 V), grayish blue in the oxidized state (1.0 V), and grayish green in highly oxidized states (1.2 V). The P(BCz-*co*-EDOT) film exhibited different colors to those of PBCz film in moderate and highly oxidized states. In similar conditions, the P(BCz-*co*-ProDOT) film was transparent in the neutral state (0.0 V), light yellowish brown in the intermediate state (0.9 V), yellowish brown in the oxidized state (1.1 V), and green in highly oxidized states (1.2 V). However, P(BCz-*co*-EDTT) film showed less multiple color variations than the P(BCz-*co*-EDOT) and P(BCz-*co*-ProDOT) films. The P(BCz-*co*-EDTT) film was transparent in the neutral state (0.0 V), gray in the intermediate state (0.8 V), and grayish brown in oxidized states (1.2 V).

**Figure 7:**
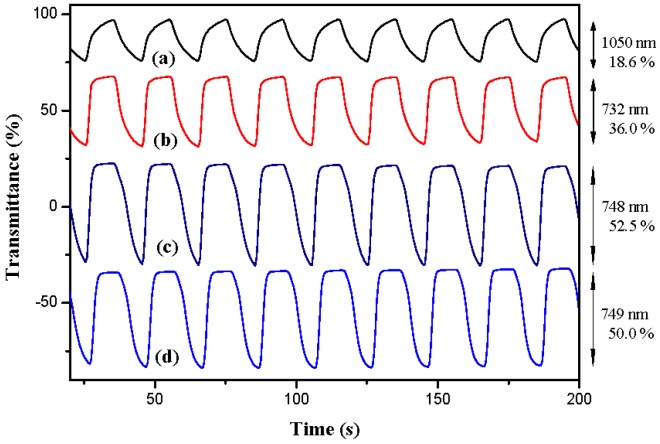
Optical contrast of: (**a**) PBCz; (**b**) P(BCz-*co*-EDOT); (**c**) P(BCz-*co*-ProDOT); and (**d**) P(BCz-*co*-EDTT) electrodes in a PC/ACN (1:1, by volume) solution containing 0.2 M LiClO_4_ between 0.0 V and 1.2 V with a residence time of 10 s.

The electrochromic switching of PBCz, P(BCz-*co*-EDOT), P(BCz-*co*-ProDOT), and P(BCz-*co*-EDTT) films at specific wavelengths were examined by double-potential-step chronoamperometry [26]. Figure 7 shows the transmittance–time profiles of PBCz, P(BCz-*co*-EDOT), P(BCz-*co*-ProDOT), and P(BCz-*co*-EDTT) films in 0.2 M LiClO_4_/(PC + ACN) solution, which were stepped by repeated potential between 0.0 and 1.2 V with a residence time of 10 s. The maximum optical contrast (Δ*T*_max_%) of PBCz, P(BCz-*co*-EDOT), P(BCz-*co*-ProDOT), and P(BCz-*co*-EDTT) from the bleaching state to the coloration state in 0.2 M LiClO_4_/(PC + ACN) solution were estimated to be 18.6%, 36.0%, 52.5% and 50.0%, respectively. Among these electrodes, P(BCz-*co*-ProDOT) film shows the highest Δ*T*_max_, and copolymers (P(BCz-*co*-EDOT), P(BCz-*co*-ProDOT), and P(BCz-*co*-EDTT)) show higher Δ*T*_max_ than that of homopolymer (PBCz) in 0.2 M LiClO_4_/(PC + ACN) solution, indicating the copolymerization of BCz with EDOT, ProDOT-Me_2_, or EDTT monomer led to an increase in the Δ*T*_max_ in 0.2 M LiClO_4_/(PC + ACN) solution.

The coloration response time (*τ*_c_) and bleaching response time (*τ*_b_) of polymer films in a solution state are summarized in Table 2; the response time was calculated at 90% of the full-transmittance change. For PBCz film in 0.2 M LiClO_4_/(PC + ACN) solution, the response time at 1050 nm was estimated to be 7.0 s from the bleaching state to the coloring state and 6.0 s from the coloring state to the bleaching state. Copolymer (P(BCz-*co*-EDOT), P(BCz-*co*-ProDOT), and P(BCz-*co*-EDTT)) films show a shorter *τ*_b_ than the PBCz film, indicating the incorporation of EDOT, ProDOT-Me_2_, or EDTT unit into the polymer backbone facilitated color change from the coloring to the bleaching state when we used 0.2 M LiClO_4_/(PC + ACN) as a supporting electrolyte.

ΔOD is the variation of optical density, which can be determined using the formula,

(1) $$\Delta \mathrm{OD}=\log\left(\frac{T_{\mathrm{ox}}}{T_{\mathrm{red}}}\right)$$

where *T*_ox_ and *T*_red_ indicate the percentage of transmittance in the oxidized state and the reduced state, respectively. The ΔOD_max_ of PBCz, P(BCz-*co*-EDOT), P(BCz-*co*-ProDOT), and P(BCz-*co*-EDTT) films in 0.2 M LiClO_4_/(PC + ACN) solution are also summarized in Table 2. Similar to the tendency of Δ*T*_max_, P(BCz-*co*-ProDOT) and P(BCz-*co*-EDTT) films show a larger ΔOD than PBCz and P(BCz-*co*-EDOT) films.

Coloration efficiency (η) is an efficient tool for the measurement of the power requirements of an electrochromic material, and can be determined using the following formula at a specific wavelength,

(2) $$\eta =\frac{\Delta \mathrm{OD}}{Q_{d}}$$

where *Q*_d_ is the injected/ejected electronic charge of the electrodes per active area, and ΔOD is the variation of optical density at a specific wavelength. As shown in Table 2, the η of PBCz film at 1050 nm, P(BCz-*co*-EDOT) film at 732 nm, P(BCz-*co*-ProDOT) at 748 nm, and P(BCz-*co*-EDTT) at 749 nm were 180.3, 78.2, 153.5, and 138.5 cm^2^∙C^−1^, respectively.

### 3.3. Spectroelectrochemistry of Electrochromic Devices (ECDs)

**Figure 8:**
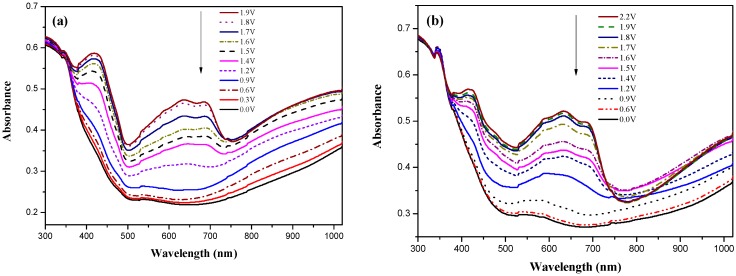

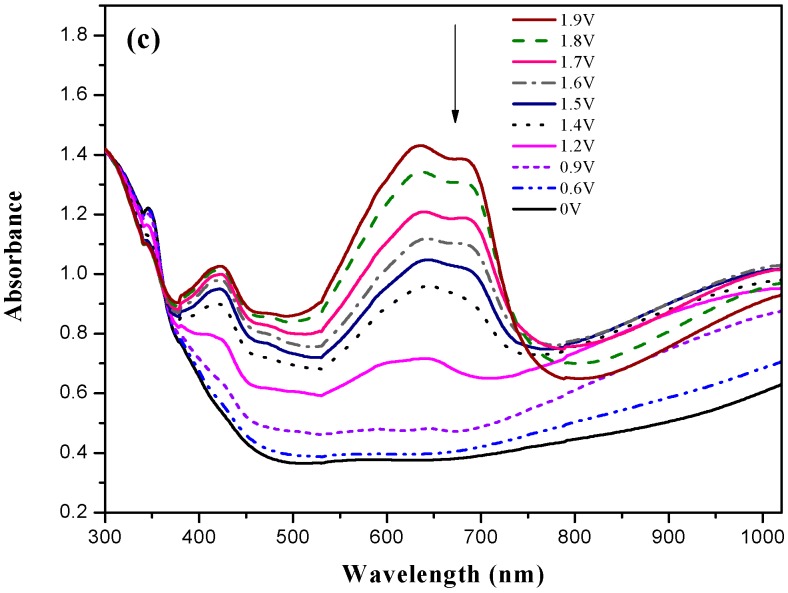
UV-Visible spectra of: (**a**) PBCz/double-layer PEDOT-PSS ECD; (**b**) P(BCz-*co*-ProDOT)/double-layer PEDOT-PSS ECD; and (**c**) P(BCz-*c*o-ProDOT)/triple-layer PEDOT-PSS ECD.

Dual type ECDs were fabricated with configurations of PBCz/double-layer PEDOT-PSS (ECD (a)), P(BCz-*co*-EDOT)/double-layer PEDOT-PSS (ECD (b)), P(BCz-*co*-ProDOT)/double-layer PEDOT-PSS (ECD (c)), P(BCz-*co*-ProDOT)/triple-layer PEDOT-PSS (ECD (c_3_)), P(BCz-*co*-ProDOT)/quadruple-layer PEDOT-PSS (ECD (c_4_)), and P(BCz-*co*-EDTT)/double-layer PEDOT-PSS (ECD (d)), and their spectroelectrochemical properties were examined by increasing the applied potential stepwise of absorbance measurements. Figure 8a–c shows the UV-Vis spectra of ECD (a), ECD (c), and ECD (c_3_), respectively. At 0.0 V, ECDs (a), (c), and (c_3_) did not reveal conspicuous absorption peak below 400 nm. At this situation, the anodically coloring material (PBCz or P(BCz-*co*-ProDOT)) was in its neutral state, revealing a light yellow color. The cathodically coloring material (PEDOT-PSS) was in its oxidized state, revealing a transparent color. The ECD (c_3_) revealed a light yellow color at 0.0 V. Upon increasing the applied potential stepwise, PBCz (or P(BCz-*co*-ProDOT)) films began to oxidize and the PEDOT-PSS layer started to reduce. Accordingly, new peaks at 420 and 640 nm emerged stepwise and the ECD (c_3_) revealed a yellowish-blue color at 1.0 V and a dark blue color at 2.0 V (Figure 1).

**Figure 9:**
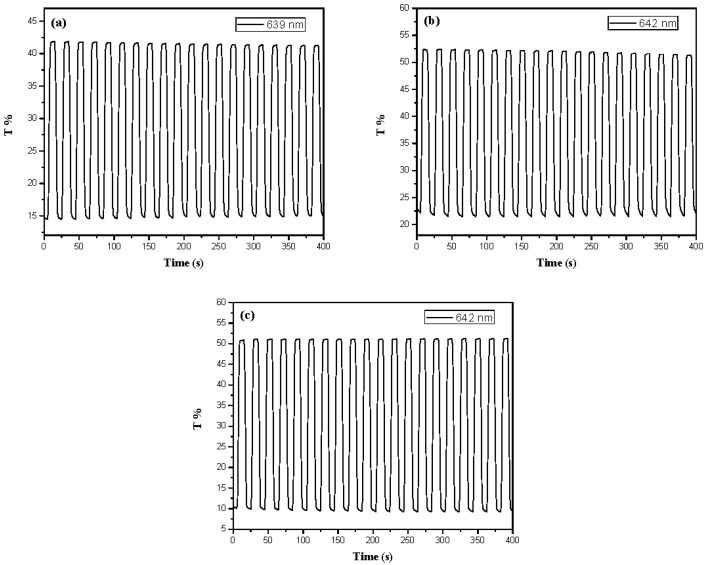
Optical contrast of (**a**) PBCz/double-layer PEDOT-PSS ECD; (**b**) P(BCz-*co*-ProDOT)/double-layer PEDOT-PSS ECD; and (**c**) P(BCz-*co*-ProDOT)/triple-layer PEDOT-PSS ECD between 0.0 V and 2.0 V with a residence time of 10 s.

Figure 9 shows the transmittance profiles as a function of time for the PBCz/double-layer PEDOT-PSS, the P(BCz-*co*-ProDOT)/double-layer PEDOT-PSS, and the P(BCz-*co*-ProDOT)/triple-layer PEDOT-PSS ECDs. The electrochromic switching of these ECDs was performed between 0.0 V and 2.0 V at a regular interval of 10 s. The Δ*T*, η, *τ*_c_, and *τ*_b_ of ECDs (a), (b), (c), (c_3_), (c_4_), and (d) estimated at 2nd double-step potential cycle are summarized in Table 3. The Δ*T* of the P(BCz-*co*-ProDOT)/double-layer PEDOT-PSS, the P(BCz-*co*-ProDOT)/triple-layer PEDOT-PSS, and the P(BCz-*co*-ProDOT)/quadruple-layer PEDOT-PSS ECDs show a larger Δ*T* than the PBCz/double-layer PEDOT-PSS ECD, indicating the incorporation of P(BCz-*co*-ProDOT) as the anodic layer led to a higher Δ*T* at 642 nm than PBCz. However, the PBCz/double-layer PEDOT-PSS ECD shows larger Δ*T* than the P(BCz-*co*-EDOT)/double-layer PEDOT-PSS and the P(BCz-*co*-EDTT)/double-layer PEDOT-PSS ECDs. Moreover, the P(BCz-*co*-ProDOT)/triple-layer PEDOT-PSS ECD showed a larger Δ*T* than the P(BCz-*co*-ProDOT)/double-layer PEDOT-PSS and the P(BCz-*co*-ProDOT)/quadruple-layer PEDOT-PSS ECDs, implying that the triple-layer PEDOT-PSS as the cathodic layer led to a higher Δ*T* than the double-layer and quadruple-layer PEDOT-PSS ECDs.

The η of ECDs (a), (b), (c), (c_3_), (c_4_), and (d) are greater than those of PBCz, P(BCz-*co*-EDOT), P(BCz-*co*-ProDOT), and P(BCz-*co*-EDTT) films in 0.2 M LiClO_4_/(PC + ACN) solution. This can be attributed to ECDs containing two polymer films (one an anodic layer, the other a cathodic layer), which are separated by an electrolyte. However, there is no complementary electrode for PBCz, P(BCz-*co*-EDOT), P(BCz-*co*-ProDOT), and P(BCz-*co*-EDTT) films. The P(BCz-*co*-ProDOT)/double-layer PEDOT-PSS, the P(BCz-*co*-ProDOT)/triple-layer PEDOT-PSS, the P(BCz-*co*-ProDOT)/quadruple-layer PEDOT-PSS, and the P(BCz-*co*-EDTT)/double-layer PEDOT-PSS ECDs show larger than that the PBCz/double-layer PEDOT-PSS ECD, indicating the incorporation of P(BCz-*co*-ProDOT) and P(BCz-*co*-EDTT) films as the anodic layers led to a higher than PBCz. However, the PBCz/double-layer PEDOT-PSS ECD shows larger than the P(BCz-*co*-EDOT)/double-layer PEDOT-PSS ECD. The *τ*_b_ of ECDs (a), (b), (c), (c_3_), (c_4_), and (d) are smaller than those of the polymer films in 0.2 M LiClO_4_/(PC + ACN) solution, indicating the ECDs changed their color faster from the doped to the dedoped state than the polymer films did in 0.2 M LiClO_4_/(PC + ACN) solution.

The comparison of Δ*T*_max_ and η with reported ECDs is shown in Table 4. The P(BCz-*co*-ProDOT)/triple-layer PEDOT-PSS ECD shows a higher Δ*T*_max_ than was reported for poly(4,4′-di(*N*-carbazoyl)biphenyl-*co*-2,2′-bithiophene)/PEDOT [27], poly(9H-carbazol-9-ylpyrene)/PEDOT [28], poly(4,4′-di(*N*-carbazolyl)biphenyl)/PEDOT [29], poly(4,4′-di(*N*-carbazoyl)biphenyl-*co*-4H-cyclopenta[2,1-b:3,4-b′]dithiophene)/PEDOT [30], poly(3,6-bis(2-(3,4-ethylenedioxy)thienyl)-*N*-methylcarbazole)/PEDOT [31], poly(2,5-bis(9-methyl-9H-carbazol-3-yl)-1,3,4-oxadiazole)/PEDOT [32], and poly(carbazole-*co*-indole-6-carboxylic acid)/PProDOT-Me_2_ ECDs [33]. In another aspect, the P(BCz-co-ProDOT)/double-layer PEDOT-PSS and the P(BCz-*co*-ProDOT)/triple-layer PEDOT-PSS ECDs show higher η at 642 nm than was reported for poly(4,4′-di(*N*-carbazoyl)biphenyl-*co*-2,2′-bithiophene)/PEDOT [27], poly(9H-carbazol-9-ylpyrene)/PEDOT [28], poly(4,4′-di(*N*-carbazoyl)biphenyl-*co*-4H-cyclopenta[2,1-b:3,4-b′]dithiophene)/PEDOT [30], and poly(carbazole-*co*-indole-6-carboxylic acid)/PProDOT-Me_2_ ECDs [33]. The high coloration efficiency of the P(BCz-*co*-ProDOT)/double-layer PEDOT-PSS and the P(BCz-*co*-ProDOT)/triple-layer PEDOT-PSS ECDs made P(BCz-*co*-ProDOT) attractive for ECD’s applications.

### 3.4. Open Circuit Memory of ECDs

**Figure 10:**
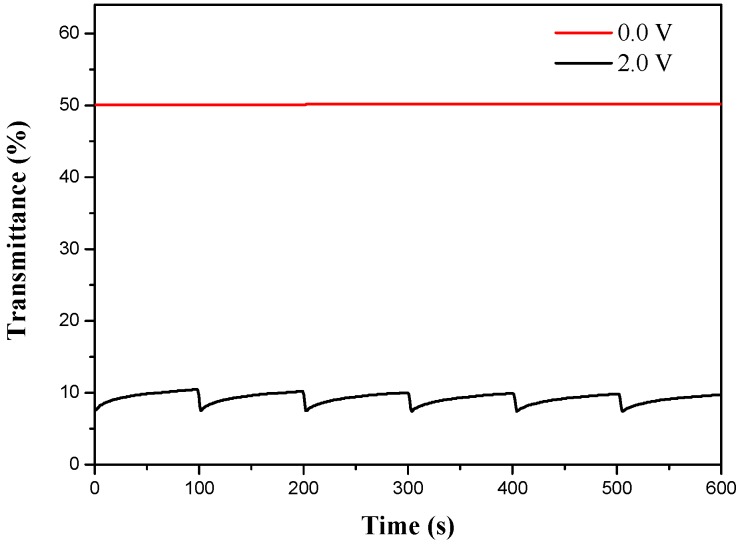
Open circuit stability of the P(BCz-*co*-ProDOT)/triple-layer PEDOT-PSS device monitored at 642 nm.

The ability to retain a colored (or bleached) state for an open circuit of the ECDs was monitored at specific wavelength as a function of time in neutral and oxidation states by applying the potential for 1 s for each 200 s time interval. As seen in Figure 10, the P(BCz-*co*-ProDOT)/triple-layer PEDOT-PSS ECD showed good optical memories in a reduced state of P(BCz-*co*-ProDOT) film, but almost no transmittance change in a reduced state. In the oxidized state of P(BCz-*co*-ProDOT) film, the ECD is rather less stable than the reduced state of P(BCz-*co*-ProDOT) film, but the transmittance change is less than 5% in an oxidized state of P(BCz-*co*-ProDOT) film, implying the P(BCz-*co*-ProDOT)/triple-layer PEDOT-PSS ECD has a reasonable optical memory under open circuit conditions.

### 3.5. Stability of Electrochromic Device (ECD)

**Figure 11:**
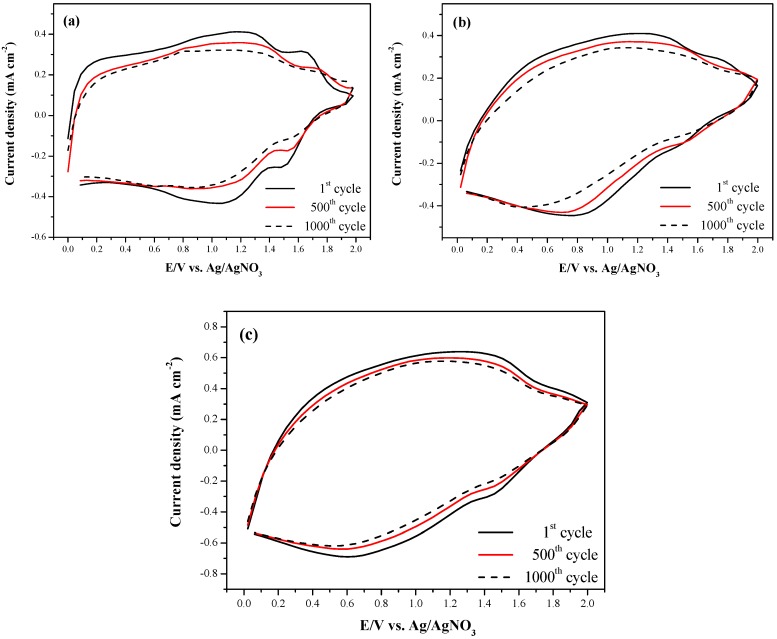
Cyclic voltammograms of: (**a**) PBCz/double-layer PEDOT-PSS ECD; (**b**) P(BCz-*co*-ProDOT)/double-layer PEDOT-PSS ECD; and (**c**) P(BCz-*co*-ProDOT)/triple-layer PEDOT-PSS ECD as a function of repeated scan with a scan rate of 500 mV∙s^−1^ between 1 and 1000 cycles.

The stability of ECD for multiple switching cycles was tested using cyclic voltammetry at the applied potentials between their oxidized and neutral states with 500 mV∙s^−1^ (Figure 11). From the observation of switching ability between oxidized and neutral states of the PBCz/double-layer PEDOT-PSS (ECD (a), Figure 11a), the P(BCz-*co*-ProDOT)/double-layer PEDOT-PSS (ECD (c), Figure 11b), and the P(BCz-*co*-ProDOT)/triple-layer PEDOT-PSS (ECD(c_3_), Figure 11c), 87%, 92%, and 92%, respectively, of electroactivity was retained after 500 cycles, and 81%, 82%, and 87%, respectively, of electroactivity was retained after 1000 cycles for ECD (a), ECD (c), and ECD (c_3_). ECD (c), which employed P(BCz-*co*-ProDOT) copolymer as an anodic layer, showed a better multiple switching stability than the PBCz homopolymer (ECD (a)), and ECD (c_3_), which employed a triple-layer PEDOT-PSS as a cathodic layer, showed a better multiple switching stability than the double layer PEDOT-PSS (ECD (c)).

## 4. Conclusions

Four anodic polymer films (PBCz, P(BCz-*co*-EDOT), P(BCz-*co*-ProDOT), and P(BCz-*co*-EDTT)) were prepared by electrochemical polymerization. The spectroelectrochemical characterizations of the anodic polymer films revealed that the P(BCz-*co*-EDOT) film was transparent in the neutral state, yellow in the intermediate state, grayish blue in the oxidized state, and grayish green in highly oxidized states. The P(BCz-*co*-ProDOT) film was transparent in the neutral state, light yellowish brown in the intermediate state, yellowish brown in the oxidized state, and green in highly oxidized states. However, the P(BCz-*co*-EDTT) film showed less multiple color variations than the P(BCz-*co*-EDOT) and P(BCz-*co*-ProDOT) films did. Electrochromic switching studies of anodic polymer films display high Δ*T*_max_ for P(BCz-*co*-ProDOT) (52.5% at 748 nm) and P(BCz-*co*-EDTT) (50.0% at 749 nm) films. Six dual type ECDs based on anodic polymer films (PBCz, P(BCz-*co*-EDOT), P(BCz-*co*-ProDOT), and P(BCz-*co*-EDTT)) and a cathodic polymer film (PEDOT-PSS) were constructed and their electrochromic behaviors were characterized. The P(BCz-*co*-ProDOT)/triple-layer PEDOT-PSS ECD revealed a light yellow color at 0.0 V, yellowish-blue color at 1.0 V, and dark blue color at 2.0 V. The P(BCz-*co*-ProDOT)/triple-layer PEDOT-PSS ECD had the highest Δ*T*_max_ (41% at 642 nm) and the P(BCz-*co*-ProDOT)/double-layer PEDOT-PSS ECD had the highest coloration efficiency (517.3 cm^2^∙C^−^^1^ at 642 nm). Moreover, the P(BCz-*co*-ProDOT)/triple-layer PEDOT-PSS ECD displayed a satisfactory optical memory property and redox stability.

## Figures and Tables

**Table 1:** Feed species of anodic polymer electrodes (a), (b), (c), and (d).

Electrodes	Anodic polymer	Feed species of anodic polymer	Feed molar ratio of anodic polymers
(a)	PBCz	2 mM BCz	Neat BCz
(b)	P(BCz-*co*-EDOT)	2 mM BCz + 2 mM EDOT	1:1
(c)	P(BCz-*co*-ProDOT)	2 mM BCz + 2 mM ProDOT-Me_2_	1:1
(d)	P(BCz-*co*-EDTT)	2 mM BCz + 2 mM EDTT	1:1

**Table 2:** Optical and electrochemical properties investigated at selected applied wavelength for the electrodes.

Electrodes	*λ* (nm) ^a^	*T*_ox_	*T*_red_	Δ*T*	ΔOD	*Q_d_* (mC∙cm^−2^)	η (cm^2^∙C^−1^)	τ_c_∙(s)	τ_b_∙(s)
(a)	1050	18.5	37.1	18.6	−0.301	−1.67	180.3	7.0	6.0
(b)	732	38.5	74.5	36.0	−0.287	−3.67	78.2	6.0	2.5
(c)	748	17.0	69.5	52.5	−0.612	−4.00	153.5	7.0	2.4
(d)	749	15.2	65.2	50.0	−0.637	−4.60	138.5	6.5	2.5

^a^ The selected applied wavelength for the electrodes.

**Table 3:** Optical and electrochemical properties investigated at selected applied wavelength for the ECDs.

Electrodes	*λ* (nm) ^a^	*T*_ox_	*T*_red_	Δ*T*	ΔOD	*Q_d_* (mC∙cm^−2^)	η (cm^2^∙C^−1^)	τ_c_ (s)	τ_b_ (s)
ECD (a)	639	14.5	41.5	27.0	−0.467	−1.45	322.1	3.0	3.0
ECD (b)	630	32.5	58.0	25.5	−0.252	−0.80	315.0	3.5	3.1
ECD (c)	642	21.5	52.5	31.0	−0.388	−0.75	517.3	3.4	2.6
ECD (c_3_)	642	10.0	51.0	41.0	−0.708	−1.70	416.5	3.0	3.0
ECD (c_4_)	642	6.1	41.1	35.0	−0.826	−2.45	337.1	3.5	2.9
ECD (d)	631	23.0	43.0	20.0	−0.272	−0.75	362.7	3.0	3.0

^a^ The selected applied wavelength for the ECDs.

**Table 4:** Electrochemical optical contrast and coloration efficiencies of carbazole-group ECDs.

ECD configuration	Δ*T*_max_ (%)	η_max_ (cm^2^∙C^−1^)	Reference
poly(4,4′-di(*N*-carbazoyl)biphenyl-*co*-2,2′-bithiophene)/PEDOT	28.6 (700 nm)	234 (700 nm)	[27]
poly(9H-carbazol-9-ylpyrene)/PEDOT	23 (623 nm)	290 (623 nm)	[28]
poly(4,4′-di(*N*-carbazolyl)biphenyl)/PEDOT	19 (550 nm)	-	[29]
poly(4,4′-di(*N*-carbazoyl)biphenyl-*co*-4H-cyclopenta[2,1-b:3,4-b′]dithiophene)/PEDOT	39.8 (628 nm)	319.98 (628 nm)	[30]
poly(3,6-bis(2-(3,4-ethylenedioxy)thienyl)-*N*-methylcarbazole)/PEDOT	ca. 30	-	[31]
poly(2,5-bis(9-methyl-9H-carbazol-3-yl)-1,3,4-oxadiazole)/PEDOT	35 (620 nm)	-	[32]
poly(carbazole-*co*-indole-6-carboxylic acid)/PProDOT-Me_2_	32 (575 nm)	372.7	[33]
P(BCz-*co*-ProDOT)/double-layer PEDOT-PSS	31 (642 nm)	517 (642 nm)	This work
P(BCz-*co*-ProDOT)/triple-layer PEDOT-PSS	41 (642 nm)	417 (642 nm)	This work
